# Explicit model predictive control for linear time-variant systems with application to double-lane-change maneuver

**DOI:** 10.1371/journal.pone.0208071

**Published:** 2018-12-04

**Authors:** Junho Lee, Hyuk-Jun Chang

**Affiliations:** 1 Department of Secured Smart Electric Vehicle, Kookmin University, Seoul, Republic of Korea; 2 School of Electrical Engineering and Department of Secured Smart Electric Vehicle, Kookmin University, Seoul, Republic of Korea; Tsinghua University, CHINA

## Abstract

Explicit model predictive control (eMPC) has been proposed to reduce the huge computational complexity of MPC while maintaining the performance of MPC. Therefore, this control method has been more widely employed in the automotive industry than MPC. In this paper, an eMPC is designed to perform a double-lane-change (DLC) maneuver. This task has been employed to demonstrate the efficacy of controllers in an autonomous driving situation. In this sense, the proposed controller shows better performance than a driver model designed in CarSim at a high vehicle longitudinal velocity. The main contribution of this paper is to present an eMPC for discrete-time linear time-variant (LTV) systems so that the proposed controller can be robust against parameter variation. In a state-space representation of the vehicle, the longitudinal velocity of the vehicle is assumed to be a constant so that the whole system is linear time-invariant (LTI). However, it is inevitable that this velocity varies in an actual driving situation. Therefore, an eMPC controller is designed using an add-on unit to consider the varying parameter without modification of the eMPC solution. The CarSim simulation results of eMPC show enhanced performance compared to that of eMPC for the LTI system.

## 1 Introduction

Model predictive control (MPC) has the ability to anticipate future events of systems and fulfill complex constraints by solving open-loop optimization problems at each sampling time [1, 2]. Owing to these advantages, MPC has been employed in various fields. In [3], a MPC scheme has been applied for improving the fuel efficiency in vehicles in a given driving situation, resulting in a significant improvement in fuel consumption. An MPC approach has also been used for charging control of electric vehicles in a smart grid [4], demonstrating that the MPC approach can improve the stability and efficiency of smart grids. In [5], nonlinear MPC has been employed for regenerative braking system of hybrid electric commercial vehicles. The hardware-in-loop test has been conducted to verify improved recovery energy. MPC has been designed in [6] for prevention of a rollover or sideslip due to variable time delays in actual driving situations. As an upper controller, the MPC controller successfully calculated the desired tire force, while fulfilling the constraints of the vehicle. Furthermore, MPC has been employed in building cooling systems in [7], where it showed the ability to successfully fulfill such constraints, and in electric circuits to reduce the capacitor voltage ripple and enhance capacitor lifetime of capacitor in [8].

However, because as MPC is based on online optimization, a significant computational complexity is inevitably caused, and this drawback limits the target application of MPC to small and/or slow problems [9]. For this purpose, explicit model predictive control (eMPC) has been suggested to reduce the computational complexity by processing the MPC scheme *offline* [10]. This offline optimization can be made available with a multi-parametric programming skill, which aims to to obtain the solution regions, the so-called critical regions, of an optimization problem by considering state variables as a vector of parameters [11]. Developments of multi-parametric programming can be found in [11, 12].

As eMPC can expand the target applications of MPC to relatively faster problems than that of online MPC, this method has been proposed in many real-time systems. Employed for adaptive cruise control, eMPC showed the suitability of the vehicle to behave in such working conditions in [13]. In [14], eMPC has been developed for real-time control of fully electric vehicles and showed improved energy efficiency by predicting a complex movement of a preceding vehicle. Moreover, eMPC has been used in electric circuits. In [15] an eMPC scheme has been suggested for DC-DC power supplies and the possibility of applying this scheme to industrial microcontrollers by taking advantage of the reduced computational complexity. In addition, an eMPC scheme has been used for active filtering and proven to be suitable for such systems, which require a large bandwidth by providing a fast response [16].

**Fig 1:**
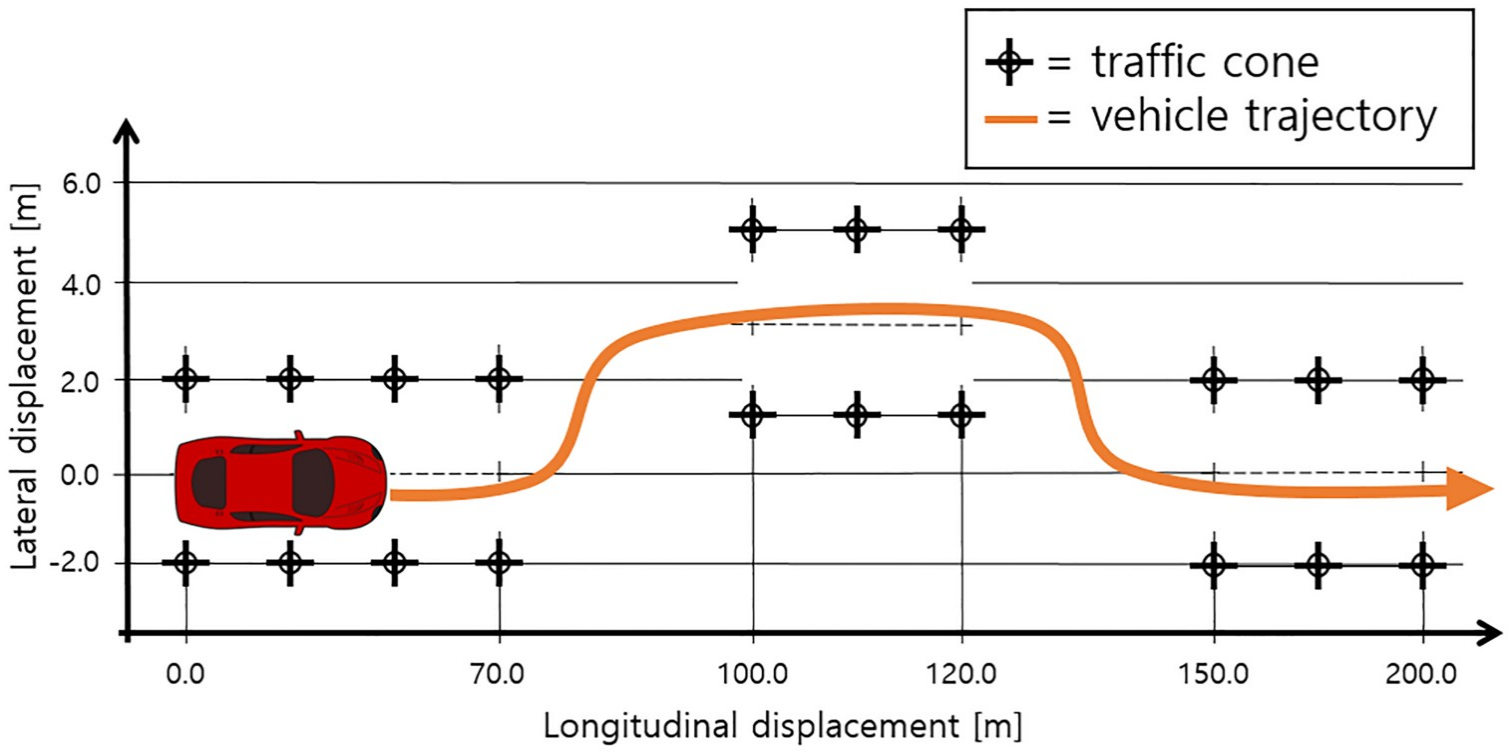
Reference road path for DLC maneuver. This path is a built-in path in CarSim, having 200 m of longitudinal distance, and the vehicle is supposed to change the lane twice within this distance. The orange line represents the trajectory of the vehicle. Performance is evaluated by checking whether the vehicle collides with the traffic cones. During driving, the longitudinal velocity of the vehicle is assumed to be constant.

This paper proposes an eMPC controller for a double-lane-change (DLC) maneuver to demonstrate the ability of the proposed controller in an autonomous driving situation. Fig 1 represents a built-in road path for a DLC maneuver in CarSim with a 30 m longitudinal range given for the lane change. The completion of the DLC maneuver can be determined by checking whether a collision with the traffic cones occurs while the vehicle is driving. DLC maneuvers have been widely used to verify the ability of vehicle controllers for path-following control. In [17], a sliding mode controller has been designed to achieve a lane change maneuver in an automated highway system. Also, modeling of a human driver by fuzzy logic has been done in [18] and verified through a DLC maneuver. For the simulation of DLC maneuvers, CarSim is used in this paper. This tool has been utilized to simulate driving tasks in order to analyze vehicle dynamics and test such vehicle controllers. Many cases using CarSim have been reported in the literature [19–22].

MPC-based controllers are mostly designed based on a discrete-time state space representation of linear time-invariant (LTI) systems. A preliminary version of the present paper, which has been published in proceedings at the *international symposium on Industrial Electronics in 2018*, also shows the design of an eMPC controller based on an LTI system. However, the parameters assumed to be constant in the LTI system change in an actual driving condition, degrading the performance of the vehicle controllers. To address this issue, we design an eMPC controller that can consider parameter changes through a few computational steps. The main concept of this approach, which has been previously reported in [23], is to compensate for errors caused in state variables due to parameter variation without modification of the critical regions of eMPC, that is, this method does not deteriorate the offline optimization framework.

For not only the steering control, but also other systems in vehicles such as transmission systmes or power management, various optimization methods for those systems have been proposed [24–26]. Therefore, the number of electric control units(ECUs) in electric vehicles has increased significantly [27]; correspondingly, the reduction of computational complexity for ECUs has been an important issue to handle. Thanks to the major advantage of eMPC, this control method can serve as a novel approach for ECUs to be capable of performing many types of vehicle control in real time. Furthermore, the eMPC controller for the LTV system in this paper has the ability to maintain the performance of MPC in terms of parameter variation caused by working conditions, aging, or degradation of systems. As a result, it can be shown that the proposed controller can perform MPC optimization offline while ensuring the stability and robustness of the controller by applying a simple add-on unit.

The remainder of this paper is organized as follows. Section 2 reviews the MPC scheme and summarizes a formulation of eMPC. Section 3 shows a state-space representation of the vehicle and the design of eMPC controller for a linear time-variant (LTV) system. The simulation results are discussed in Section 4, and the conclusion and future work are presented in Section 5 and 6.

## 2 Explicit model predictive control

Based on the receding policy, MPC solves an open-loop optimization problem (1) at each sampling time.

$$\begin{array}{c} \begin{array}{cc} & \underset{U≜u_{t},\ldots ,u_{t+N_{u}-1}}{min}\{J\left(U,x_{t}\right)=x_{t+N_{y}|t}^{T}Px_{t+N_{y}|t}+\\ & \;\;\;\;\;\;\;\;\;\;\;\;\;\;\sum_{k=0}^{N_{y}-1}[x_{t+k|t}^{T}Qx_{t+k|t}+u_{t+k}^{T}Ru_{t+k}\\ & \;\;\;\;\;\;\;\;\;\;\;\;\;\;+{\left(y_{t+k}-y_{t+k}^{ref}\right)}^{T}QR\left(y_{t+k}-y_{t+k}^{ref}\right)]\},\\ \text{s}.\text{t}.\; & u_{min}\leq u_{t+k|t}\leq u_{max},\;k=0,1,\ldots ,N_{u},\\ & x_{min}\leq x_{t+k|t}\leq x_{max},\;k=0,1,\ldots ,N_{y},\\ & x_{t|t}=x_{t},\\ & x_{t+k+1|t}=A_{d}x_{t+k|t}+B_{d}u_{t+k},\;k⩾0,\\ & y_{t+k|t}=C_{d}x_{t+k|t},\\ & u_{t+k|t}=Kx_{t+k|t},\;N_{u}<k\leq N_{y}, \end{array} \end{array}$$ (1)

where *A*_*d*_, *B*_*d*_, and *C*_*d*_ are, respectively, the system, input, and output matrix in discrete-time. *k* is the time index, and *x*_*t*+*k*|*t*_ indicates the predicted state vector at time *t* + *k* from the current time *t*. *N*_*u*_ and *N*_*y*_ denote the prediction horizon for the state vector *x*_*t*_ and input horizon for the input vector *u*(*t*). It is assumed that *Q* = *Q*^*T*^ ≽ 0, *R* = *R*^*T*^ ≻ 0, *QR* = *QR*^*T*^ ≽ 0, and *P* ≽ 0. The matrix *P* for the predicted state vector at *N*_*y*_ and the gain matrix *K*, the control law for *N*_*u*_ < *k* ≤ *N*_*y*_, are the solution of the discrete-time algebraic Riccati equation (ARE) as follows:

$$\begin{array}{c} \begin{array}{cc} & P=A_{d}^{T}PA_{d}-A_{d}^{T}PB_{d}K+Q=0,\\ & K={\left(B_{d}^{T}PB_{d}+R\right)}^{-1}B_{d}^{T}PA_{d}. \end{array} \end{array}$$ (2)

Based on these weighting matrices, the MPC controller obtains a sequence of optimal vectors *U*, which minimizes the cost function *J*. Then, the controller applies the first input vector *u*_*t*_ to the system while discarding others. As *u*_*t*_ is obtained considering the constraints over *N*_*y*_ and *N*_*u*_, MPC can anticipate future events of the system and fulfill such complex constraints.

Despite these advantages, a considerable computational load due to online optimization of MPC has been regarded as a huge drawback. Therefore, a new MPC scheme, which can reduce the computational burden, has been suggested in [10]. Called explicit MPC, this approach uses a multi-parametric quadratic programming (mp-QP) skill to perform MPC offline.

By using the prediction equation of the state vector as follows

x(t)=Φx(t)+Λu(t),

where

$$\begin{array}{l} \text{x}(t)=\left[\begin{array}{c} x_{k+1|k}\\ \vdots \\ x_{k+N_{y}|k} \end{array}\right],{\text{u}}_{t}=\left[\begin{array}{c} u_{t}\\ \vdots \\ u(k+N_{y}-1|k) \end{array}\right],\\ \Phi =\left[\begin{array}{c} A_{d}\\ \vdots \\ A_{d}^{N_{y}} \end{array}\right],\;\Lambda =\left[\begin{array}{cccc} B_{d} & 0 & \cdots & 0\\ A_{d}B_{d} & B_{d} & \cdots & 0\\ \vdots & \vdots & \ddots & \vdots \\ A_{d}^{N_{y}-1}B_{d} & A_{d}^{N_{y}-2}B_{d} & \cdots & B_{d} \end{array}\right], \end{array}$$ (3)

the optimization problem (1) can be reformulated as follows:

$$\begin{array}{c} \begin{array}{cc} & J\left(U,x_{t}\right)=U^{T}HU+2x_{t}^{T}FU+x_{t}^{T}Gx_{t},\\ & \text{s}.\text{t}.\;\Phi_{c}U\leq B_{c}+A_{c}x_{t},\\ & \text{where}\\ & H=\Phi^{T}\hat{Q}\Phi +\hat{R},\;F=\Phi^{T}\hat{Q}\Lambda ,\;G=\Lambda^{T}\hat{Q}\Lambda +Q,\\ & \Phi =[\begin{array}{c} \Phi_{i}\\ -\Phi_{i} \end{array}],\;i=1,\ldots ,N_{y},\;B_{c}=[\begin{array}{c} x_{max}\\ -x_{min} \end{array}],\\ & A_{c}=[\begin{array}{c} -A_{d}^{i}\\ A_{d}^{i} \end{array}],\;i=1,\ldots ,N_{y},\\ & \hat{Q}=[\begin{array}{cccc} Q & & & \\ & \ddots & & \\ & & Q & \\ & & & P \end{array}],\;\hat{R}=[\begin{array}{ccc} R & & \\ & \ddots & \\ & & R \end{array}]. \end{array} \end{array}$$ (4)

The matrices *H*, *F*, and *G* can be obtained offline, and Φ_*i*_ indicates the *i*th row of Φ.

From (4), an mp-QP problem can be formulated by defining $z≜U+H^{-1}F^{T}x_{t}$ as follows:

$$\begin{array}{l} J_{z}\left(x_{t}\right)=\underset{z}{\text{min}}\frac{1}{2}z^{T}Hz\\ \text{s}.\text{t}.\;Wz\leq G+Sx_{t}, \end{array}$$ (5)

where $J_{z}\left(x_{t}\right)=J\left(U,x_{t}\right)-\frac{1}{2}x_{t}^{T}\left(Y-FH^{-1}F^{T}\right)x_{t}$, $S≜C_{c}+GH^{-1}F^{T}$, and *H* is a weighting matrix for *z*. The inequality constraint in (5), which is defined by *x*_*t*_, is a polytope, which means the generated critical regions are also polytope. The optimization variable *z* is a continuous piecewise affine; correspondingly, the optimal input *u*_*t*_ is also a continuous piecewise affine function of *x*_*t*_ [10]. One approache to forming the critical regions has been suggested in [28] as

$$\begin{array}{c} \begin{array}{cc} CR_{1} & =C_{j}.A<C_{j}.b,\;j=1,\ldots ,q,\\ CR_{j+1} & =\{C_{j}.A>C_{j}.b\;\text{and}\;C_{j}.A\leq C_{j}.b,\;\forall i<j\},\;j=1,\ldots ,q, \end{array} \end{array}$$ (6)

where *CR*_*j*_, *C*_*j*_, and *q* indicate the *j*th critical region, inequality constraint, and the number of constraints in the mp-QP problem (5) respectively. The whole space of the parameter vector *z* is the sum of the critical regions. In this paper, the parametric optimization (POP) toolbox from the PAROC Platform is used [29]. On the basis of the values of *z*, the eMPC controller *explicitly* chooses a critical region among the critical regions. A unique sequence of MPC laws is contained in each critical region so that the controller can obtain a sequence of the optimal vector *U* in (1). Then, as MPC processes, only the first input vector *u*_*t*_ is chosen as the actual control input to the system. Note that in the eMPC scheme, the task of solving the complicated optimization problem (1) at each sampling time turns into a simple computational step of choosing a critical region.

## 3 Controller design

### 3.1 Vehicle dynamics

In this subsection, the vehicle dynamics and a state-space representation reflecting a varying parameter will be addressed.

**Fig 2:**
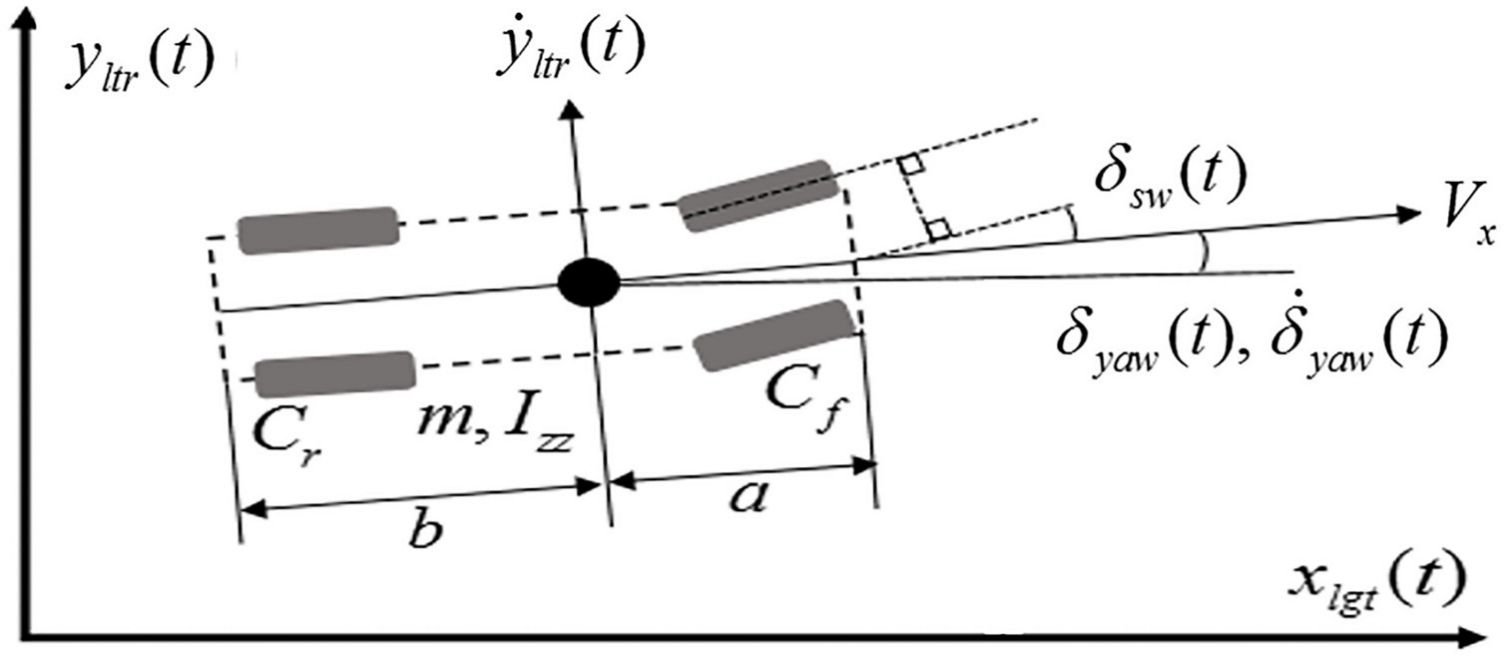
Vehicle dynamics. Assuming that the longitudinal velocity is constant, the two degrees of freedom are represented by the lateral displacement, *y*_*ltr*_(*t*), and the yaw angle *δ*_*yaw*_(*t*). The values of the parameters can be found in Table 1.

Fig 2 represents a vehicle model with longitudinal displacement *x*_*lgt*_(*t*) and lateral displacement *y*_*ltr*_(*t*). Table 1 lists the vehicle parameters, where its values refer to a vehicle model in CarSim, C-Class Hatchback 2017. *x*_*lgt*_(*t*) and *y*_*ltr*_(*t*) can be obtained as follows:

$$\begin{array}{c} \begin{array}{cc} & {\dot{x}}_{lgt}\left(t\right)=V_{x}\text{cos}\delta_{yaw}\left(t\right)-{\dot{y}}_{ltr}\left(t\right)\text{sin}\delta_{yaw}\left(t\right)\\ & {\dot{y}}_{ltr}\left(t\right)=V_{x}\text{sin}\delta_{yaw}\left(t\right)+{\dot{y}}_{ltr}\left(t\right)\text{cos}\delta_{yaw}\left(t\right), \end{array} \end{array}$$ (7)

where *δ*_*yaw*_(*t*) is the yaw angle of the vehicle. Considering that ${\dot{y}}_{ltr}\left(t\right)$ and *δ*_*yaw*_(*t*) in (7) is significantly smaller than *V*_*x*_ in DLC maneuvers [30], (7) can be simply linearized as follows:

$$\begin{array}{c} \begin{array}{cc} & {\dot{x}}_{lgt}\left(t\right)=V_{x}\\ & {\dot{y}}_{ltr}\left(t\right)=V_{x}\delta_{yaw}\left(t\right)+{\dot{y}}_{ltr}\left(t\right). \end{array} \end{array}$$ (8)

The motion equation of *y*_*ltr*_(*t*) and *δ*_*yaw*_(*t*) of the vehicle can be presented as follows [30]

$$\begin{array}{cc} m{\ddot{y}}_{ltr}\left(t\right)= & -\frac{\left(C_{f}+C_{r}\right)}{V_{x}}{\dot{y}}_{ltr}\left(t\right)\\ & -\{\frac{aC_{f}+bC_{r}}{V_{x}}+mV_{x}\}{\dot{\delta }}_{yaw}\left(t\right)+\frac{C_{f}}{G}\delta_{sw}\left(t\right),\\ I_{zz}{\ddot{\delta }}_{yaw}\left(t\right)= & -\frac{\left(aC_{f}-bC_{r}\right)}{V_{x}}{\dot{y}}_{ltr}\left(t\right)\\ & -\frac{a^{2}C_{f}+b^{2}C_{r}}{V_{x}}{\dot{\delta }}_{yaw}\left(t\right)+\frac{aC_{f}}{G}\delta_{sw}\left(t\right). \end{array}$$ (9)

In an actual driving situation, it is nearly impossible for the driver to maintain a constant longitudinal velocity *V*_*x*_. Therefore, in this paper, *V*_*x*_ is considered a varying parameter, *V*_*x*_(*t*).

**Table 1:** Parameters of vehicle model.

Symbol	Description	Value (units)
*V*_*x*_	longitudinal vehicle velocity	m/s
*m*	vehicle mass	1270 kg
*I*_*zz*_	vehicle yaw inertia	1995.78 kg⋅m^2^
*a*	distance from center to front axis	1.192 m
*b*	distance from center to rear axis	1.548 m
*C*_*f*_	front tire stiffness	55405 N/rad
*C*_*r*_	rear tire stiffness	55405 N/rad
*G*	average steering ratio	15.29

Using (9), the state-space representation of the LTV system can be obtained as follows:

$$\begin{array}{c} {\dot{x}}_{t}=A\left(t\right)x_{t}+Bu_{t}\\ y_{t}=Cx_{t}, \end{array}$$ (10)

where

$$\begin{array}{c} \begin{array}{cc} & \;\;\;\;\;\;\;\;\;\;\;x_{t}\in R^{n},\;u_{t}\in R^{m},\\ & A\left(t\right)=[\begin{array}{cccc} -\frac{\left(C_{f}+C_{r}\right)}{mV_{x}\left(t\right)} & -\frac{aC_{f}-bC_{r}}{mV_{x}\left(t\right)}-V_{x}\left(t\right) & 0 & 0\\ -\frac{aC_{f}-bC_{r}}{I_{zz}V_{x}\left(t\right)} & -\frac{a^{2}C_{f}+b^{2}C_{r}}{I_{zz}V_{x}\left(t\right)} & 0 & 0\\ 1 & 0 & 0 & V_{x}\left(t\right)\\ 0 & 1 & 0 & 0 \end{array}],\\ & B=[\begin{array}{c} \frac{C_{f}}{mG}\\ \frac{aC_{f}}{I_{zz}G}\\ 0\\ 0 \end{array}],\;C=[\begin{array}{cccc} 0 & 0 & 1 & 0 \end{array}]. \end{array} \end{array}$$ (11)

In (10), $x_{t}=[\begin{array}{cccc} {\dot{y}}_{ltr}\left(t\right) & {\dot{\delta }}_{yaw}\left(t\right) & y_{ltr}\left(t\right) & \delta_{yaw}\left(t\right) \end{array}{]}^{T}$ and *u*(*t*) = *δ*_*sw*_(*t*). The continuous-time state-space representation (10) is discretized with a sampling time of 0.01 s as follows:

$$\begin{array}{c} x\left[k+1\right]=A_{d}\left[k\right]x\left[k\right]+B_{d}u\left[k\right]\\ y\left[k\right]=C_{d}x\left[k\right], \end{array}$$ (12)

where

$$\begin{array}{cc} & A_{d}\left[k\right]=[\begin{array}{cccc} 0.9676 & A_{d}^{1}\left[k\right] & 0 & 0\\ 0.0109 & A_{d}^{2}\left[k\right] & 0 & 0\\ 0.0098 & 0.0001 & 1 & A_{d}^{3}\left[k\right]\\ A_{d}^{4}\left[k\right] & 0.0097 & 0 & 1 \end{array}],B_{d}=[\begin{array}{c} 0.0250\\ 0.0234\\ 0.0001\\ 0.0001 \end{array}],\\ & C_{d}=[\begin{array}{cccc} 0 & 0 & 1 & 0 \end{array}], \end{array}$$ (13)

where $A_{d}^{i}\left[k\right]$, *i* = 1, …, 4 indicates the varying elements due to *V*_*x*_(*t*), and *k* is the sampling time index.

### 3.2 Explicit MPC for LTI system

First, the design of the eMPC controller with the LTI system, which treats the varying parameter *V*_*x*_(*t*) as a constant value, will be presented.

The constraints of the states and input are determined as follows:

$$\begin{array}{c} \begin{array}{cc} & [\begin{array}{c} -0.65\\ -25.21\\ -1\\ -14.32 \end{array}]\leq [\begin{array}{c} {\dot{y}}_{ltr}\left(t\right)\;\left[\text{km/h}\right]\\ {\dot{\delta }}_{yaw}\left(t\right)\;\left[\text{deg/s}\right]\\ y_{ltr}\left(t\right)\;\left[\text{m}\right]\\ \delta_{yaw}\left(t\right)\;\left[\text{deg}\right] \end{array}]\leq [\begin{array}{c} 0.65\\ 25.21\\ 4.5\\ 14.32 \end{array}],\\ & \;\;\;\;\;\;-100\leq \delta_{sw}\left(t\right)\;\left[\text{deg}\right]\leq 100. \end{array} \end{array}$$ (14)

The constraints of ${\dot{y}}_{ltr}\left(t\right)$ and *δ*_*yaw*_(*t*) are set to satisfy the assumption applied in (8). Other constraints are determined based on the boundaries of the driver model states in CarSim, with a longitudinal velocity of 60 km/h and the geometrical constraint of the road path.

The determination of the weighting matrices in (1) is set to

$$\begin{array}{c} Q=[\begin{array}{cccc} 0.04 & 0 & 0 & 0\\ 0 & 0.002 & 0 & 0\\ 0 & 0 & 0.001 & 0\\ 0 & 0 & 0 & 125 \end{array}],R=1\times 10^{-6},QR=35. \end{array}$$ (15)

As the value of the weighting factor increases, the associated state or input is more dominantly considered in solving (1). The values in (15) are chosen with respect to this relation through trial and error. The matrix *P* in (1), which acts as the weighting matrix for the terminal states, is given by the discrete-time Riccati Eq (2).

**Fig 3:**
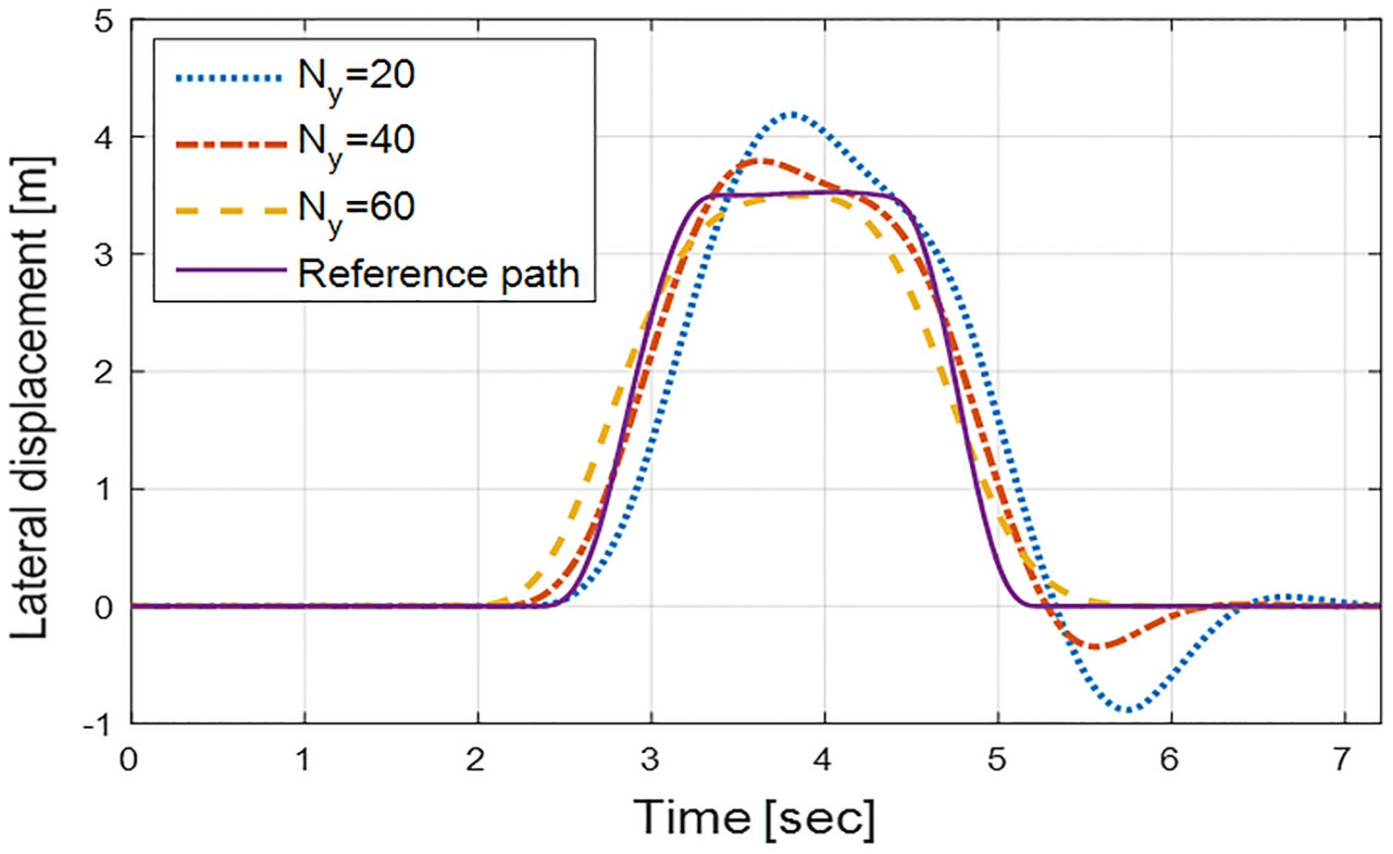
Lateral displacement of the vehicle with different prediction horizons. This figure shows the tracking ability of the proposed controller with prediction horizons of 20, 40, and 60. When the prediction horizon is 20, a relatively large error in the lateral displacement appears as the prediction ability of the controller is degraded. On the other hand, when the prediction ability increases overwhelmingly a prediction horizon of 60 in this case, an extremely early steering is observed. Therefore, the prediction horizon is set to 40 for the controller.

The input horizon *N*_*u*_ is set to 1, and to obtain the range of the prediction horizon *N*_*y*_, some cases of *N*_*y*_ are set and simulated as shown in Fig 3. It can be found that the ability of eMPC controllers to predict future events, which is *y*_*ltr*_(*t*) in this case, is enhanced with a long range of *N*_*y*_. However, in this simulation, an excessively long range of *N*_*y*_ results in an unreasonably earlier steering, which can be seen in the case of *N*_*y*_ = 60 as well as increasing the number of critical regions which consumes a large memory size. Because this control action may cause the vehicle to collide with the traffic cones, *N*_*y*_ is set to 40 in this paper.

**Fig 4:**
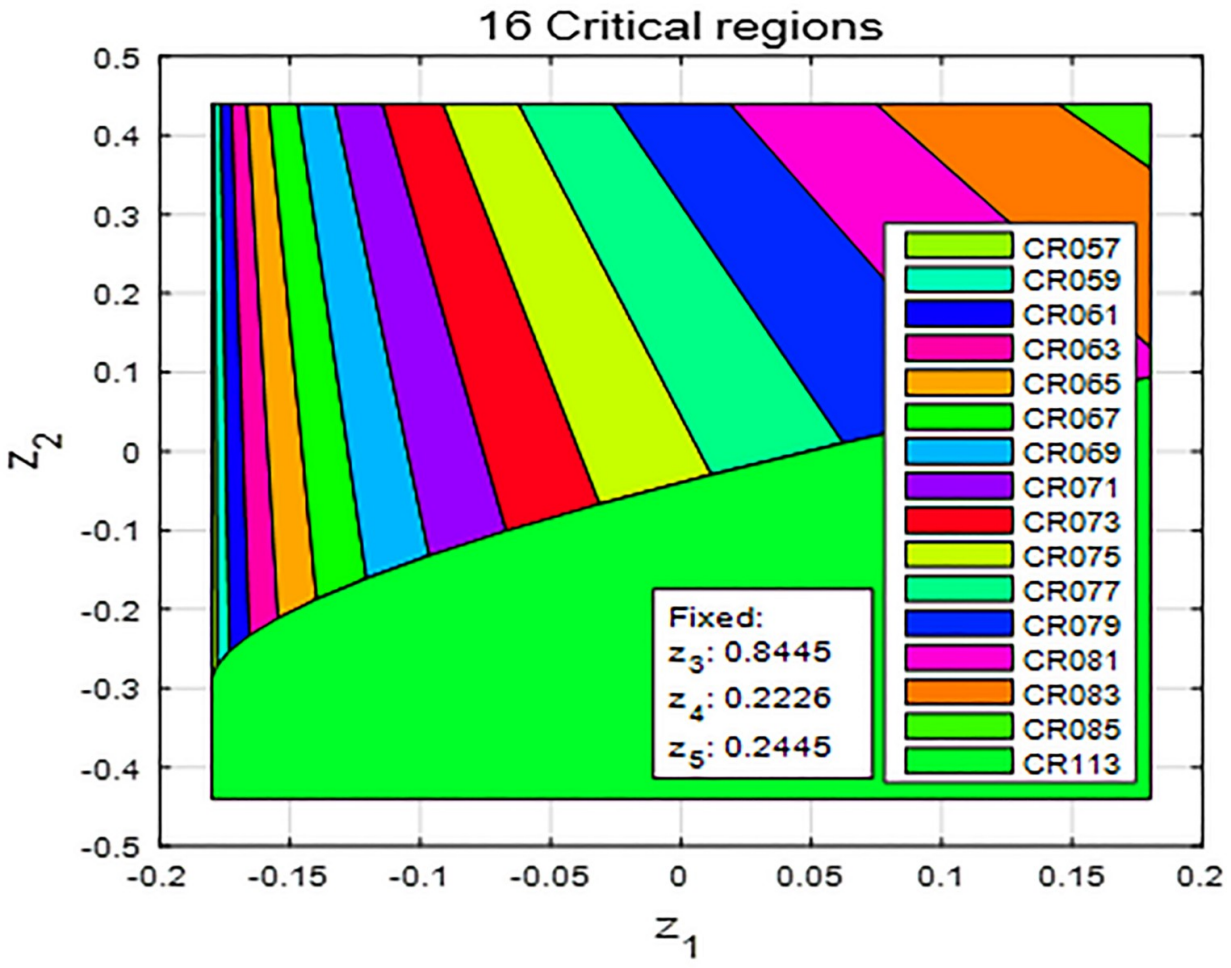
Critical regions. The generated critical regions of the eMPC controller can be seen in this figure, where $\left[z_{1},\ldots ,z_{5}]=[{\dot{y}}_{ltr}(t)\;{\dot{\delta }}_{yaw}(t)\;y_{ltr}(t)\;\delta_{yaw}(t)\;y_{ltr}^{ref}(t)\right]$; 113 critical regions were formed based on the constraint set, of which 16 regions are feasible for obtaining an optimal control input when only *z*_1_ and *z*_2_ vary and the other parameters are fixed. Each region owns its unique sequence of optimal vectors, and the controller *explicitly* chooses a region based on the parameter values.

A total of 113 critical regions were generated using the POP toolbox and Fig 4 shows several of feasible critical regions where only two parameters, *z*_1_ and *z*_2_, vary in the parameter vector *z*. In this figure, $\begin{array}{c} \left[z_{1},\ldots ,z_{5}\right] \end{array}=\begin{array}{ccccc} {}[{\dot{y}}_{ltr}\left(t\right) & {\dot{\delta }}_{yaw}\left(t\right) & y_{ltr}\left(t\right) & \delta_{yaw}\left(t\right) & y_{ltr}^{ref}\left(t\right)] \end{array}$. The range where *z*_1_ and *z*_2_ are bounded by the constraint set (14). Each critical region owns its unique sequence of optimal vectors and a region is chosen based on the values of *z*_1_ and *z*_2_. The eMPC controller selects the region by simply calculating inequality constraints instead of solving the optimization problem (1) at each sampling time. This reduction in computational load relieves the burden of implementing industrial microcontrollers in real-time situations [31]. For an actual implementation of eMPC controllers, the POP toolbox forms critical regions in the controller design process as in this subsection. Then, eMPC controllers employ the conditional statement written by C language, which represents generated critical regions. Based on the values of the variables, controllers explore this statement to obtain an optimal input vector for the real-time closed-loop control.

An explicit MPC controller with an LTI system, where *V*_*x*_ is assumed to be constant, is designed and compared with an LTV system, which will be addressed in Section 3.3.

### 3.3 Explicit MPC for LTV systems

Considering an actual driving situation, the LTI system matrix *A*_*d*_ is changed into the time-variant system matrix *A*_*d*_[*k*] as determined in (13). In this paper, *A*_*d*_ is denoted by ${\hat{A}}_{d}$, which we assume to be known as we know the initial value.

In this subsection, an algorithm proposed [23] to compensate the state vector *x*[*k*] will be presented. Through this algorithm, the eMPC controller can be made robust against parameter changes.

**Fig 5:**
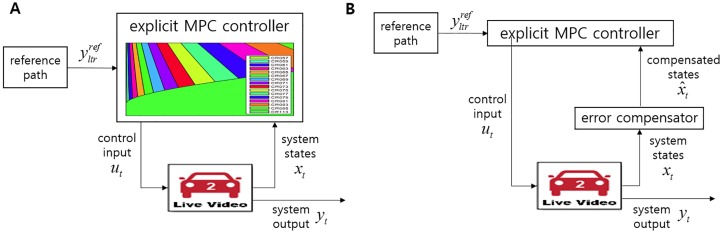
Frameworks of eMPC for LTI systems (A) and LTV systems (B). In terms of parameter variation, there is no alternative way in Fig 5A to adjust the variation because the critical regions cannot be changed with respect to variation. In contrast, in Fig 5B, compensating for the state vector with an error compensator, enables the controller to be robust against such parameter variation. The main advantage of this approach is that, by simply adding a compensator, where only matrix multiplication is taken in its process, no modification of the critical regions is necessary.

Fig 5A illustrates the implementation of eMPC for LTI systems. Based on the critical regions, which are formed by LTI systems, and the measured state vector *x*[*k*], the eMPC controller explicitly obtains an optimal input *u*[*k*]. Because the eMPC controller is designed based on ${\hat{A}}_{d}$, the controller is vulnerable to such parameter variation. Whereas Fig 5B shows the implementation of eMPC, where an error compensator, which is a simple linear matrix, is added to compensate for the state vector according to the variation in the system matrix.

The matrix *A*_*d*_[*k*] can be determined as

$$A_{d}\left[k\right]={\hat{A}}_{d}+\sum_{i=1}^{N_{v}}\Delta_{i}\alpha_{i}\left[k\right],$$ (16)

where $\Delta_{i}\in R^{n\times n}$, $\alpha_{i}\left(t\right)\in R$, and *N*_*v*_ is the number of varying parameters. We know which elements vary but how the matrix evolves is unknown.

Eq (12) can be rewritten using (16) as follows:

$$\begin{array}{c} \begin{array}{cc} x\left[k\right]-{\hat{A}}_{d}x\left[k-1\right]-B_{d}u\left[k-1\right] & =\sum_{i=1}^{N_{v}}\Delta_{i}\alpha_{i}\left[k-1\right]x\left[k-1\right],\\ & =D\left[k-1\right]\left[\alpha_{1}\left[k-1\right]\right),\alpha_{1}\left[k-1\right]),\\ & \;\;\;\;\;\ldots ,\alpha_{N_{v}}\left[k-1\right]], \end{array} \end{array}$$ (17)

where $D\left[k-1\right]=[\Delta_{1}x\left[k-1\right],\Delta_{2}x\left[k-1\right],\ldots ,\Delta_{N_{v}}x\left[k-1\right]]$ and $\Delta_{i}x\left[k-1\right]\in R^{n}$, *i* = 1, …, *N*_*v*_; thus $D\left[k-1\right]\in R^{n\times m}$.

In (17), the previous states, *x*[*k* − 1], and input, *u*[*k* − 1], are known by measurement. Based on the Rouché–Capelli theorem, (17) has a unique solution *α*_*i*_[*k* − 1], *i* = 1, …, *N*_*v*_ for all t≥1.

As a result, by substituting (17) into (16), *A*_*d*_[*k* − 1] can be obtained based on the recent history of the states and input, and the current state measurement. In order to obtain the gain matrix *L*[*k*] for the compensation of the state vector, the following equation can be considered:

$$\hat{x}\left[k+1\right]={\hat{A}}_{d}\hat{x}\left[k\right]+B_{d}u\left[k\right],$$ (18)

where $\hat{x}\left[k\right]$ denotes a compensated state vector at the time instant *k*. Assume that the initial system matrix ${\hat{A}}_{d}$ is invertible, and $\hat{x}\left[k\right]$ in (18) is replaced by *L*[*k*]*x*[*k*], where

$$L\left[k\right]={\hat{A}}_{d}^{-1}A_{d}\left[k\right].$$ (19)

Then, considering (12) and (18), $\hat{x}\left[k+1\right]$ = *x*[*k* + 1], which means the compensated state vector can be identical with the state vector obtained by the time varying system matrix *A*_*d*_[*k*] in (12) by employing the gain matrix in (19).

By substituting *A*_*d*_[*k* − 1] from (17) for *A*_*d*_[*k*] in (19), *L*[*k* − 1] can be calculated. Then, with sufficiently slow time-varying parameters, it can be assumed that *L*[*k*] ≅ *L*[*k* − 1]; consequently, the state vector can be compensated as follows:

$$\hat{x}\left[k\right]=L\left[k\right]x\left[k\right]≅L\left[k-1\right]x\left[k\right].$$ (20)

Because (20), the compensation of the state vector in Fig 5B, is a simple matrix multiplication based on the previous states and input, this compensator does not need modification of the critical regions of eMPC regarding parameter variation. For the better understanding, a full explanation about this approach can be found in [23].

## 4 Simulation results

**Fig 6:**
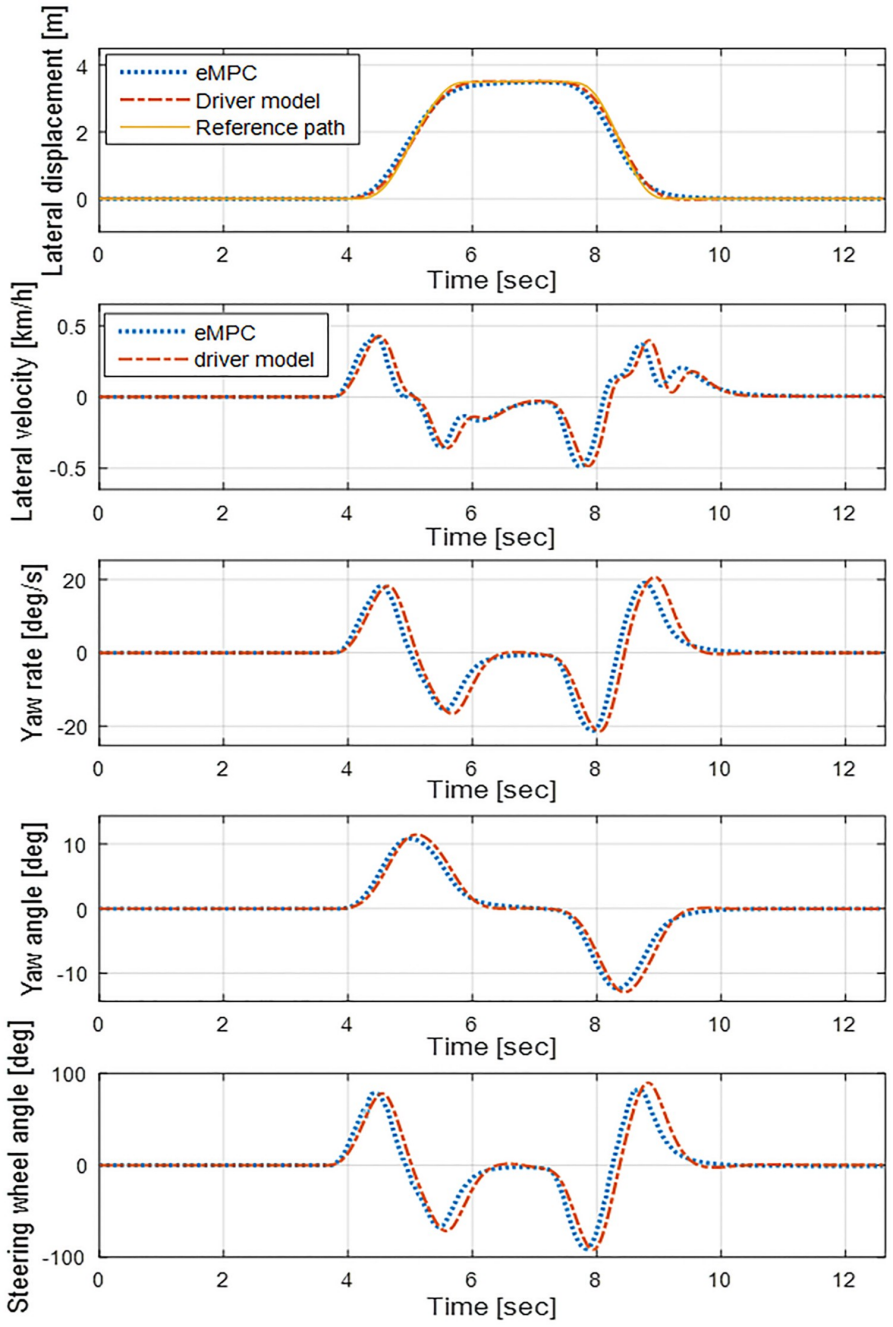
Simulation result of eMPC controller for the LTI system at a longitudinal velocity of 60 km/h. This figure shows the fulfillment of the constraint set (14) of the eMPC controller. The vertical range of each window is identical with the constraints of each variable and input.

Fig 6 shows the simulation results of the eMPC controller for an LTV system at 60 km/h longitudinal velocity, where the driver model is a built-in model in CarSim. The plot of the lateral displacement shows that both eMPC controllers and the driver model succeed in the DLC maneuver. This figure also demonstrates the fulfillment of the constraints of the eMPC controller by showing that all variables are within the boundaries.

**Fig 7:**
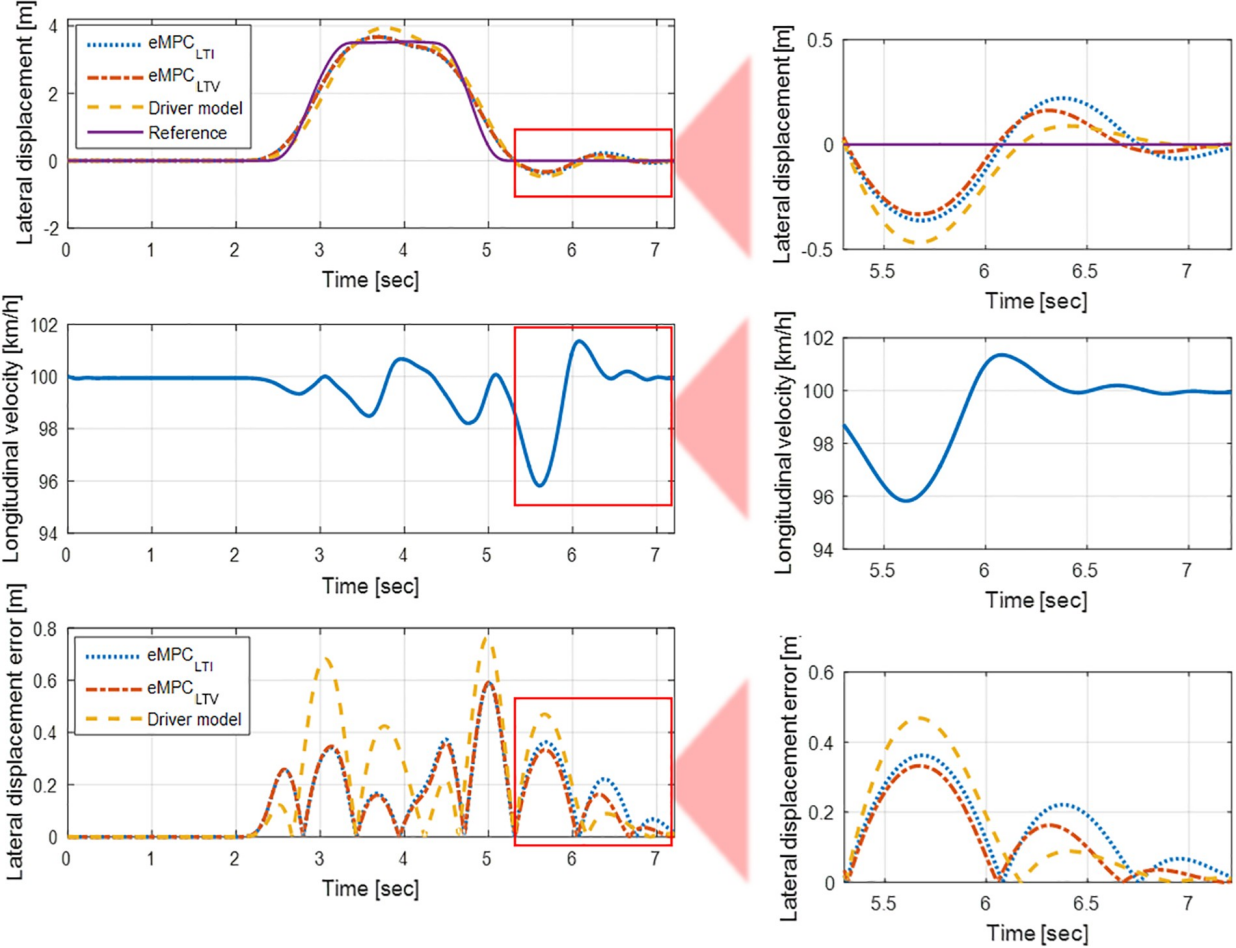
Lateral displacement of eMPC controllers for LTI and LTV systems with driver model at 100 km/h longitudinal velocity. The longitudinal velocity of the vehicle varies during the DLC maneuver despite the fact that this value is intended to be constant. An improved performance of the eMPC controller for the LTV system can be found in the lateral displacement error. After the longitudinal velocity starts varying, the error decreases compared to that of the eMPC controller for the LTI system.

**Fig 8:**
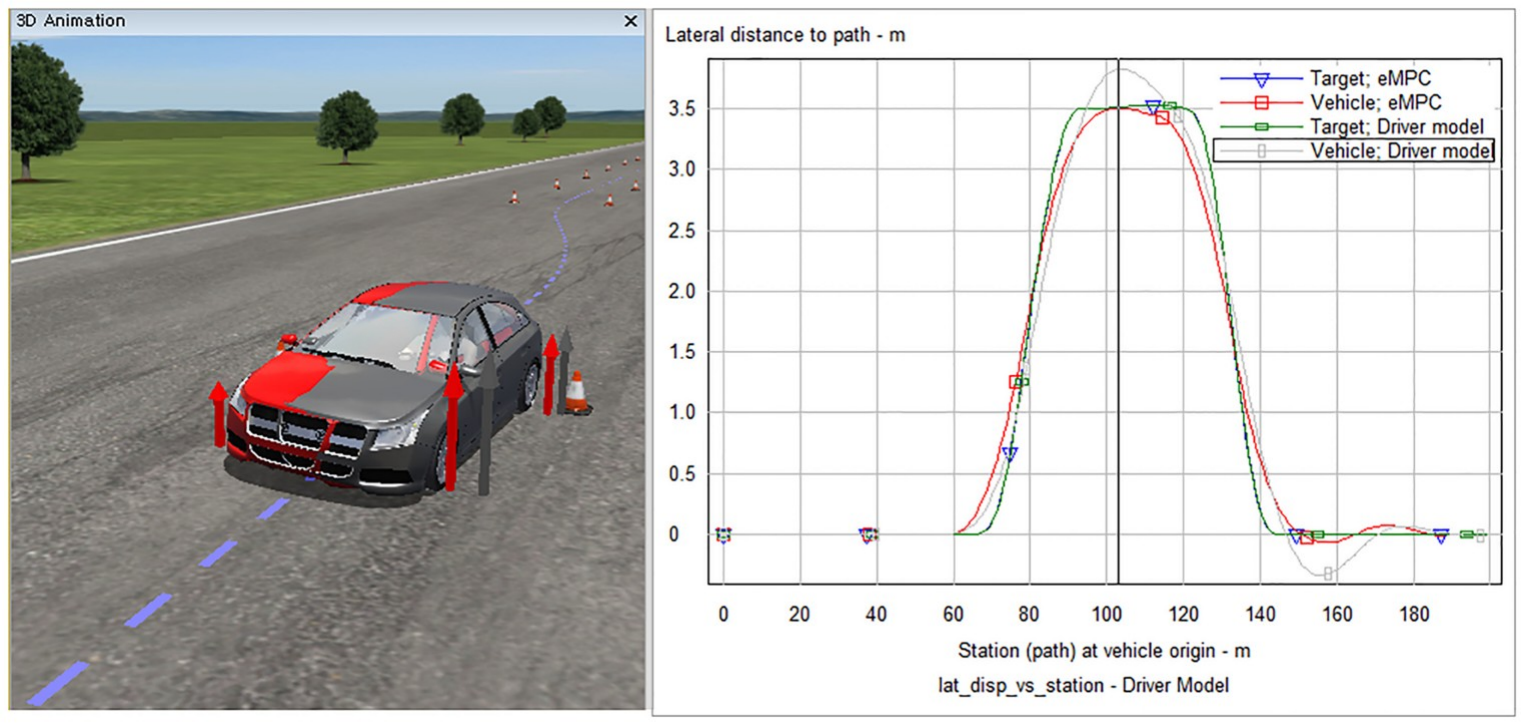
CarSim simulation of proposed controller and driver model. This figure shows the lateral displacement of the proposed controller and the driver model in CarSim. At a high longitudinal velocity (100 km/h here), the performance of the driver model is deteriorated and nearly causes a collision with a traffic cone. In contrast, the proposed controller maintains a reasonable distance away from the traffic cones.

Fig 7 shows the simulation results of eMPC controllers for both LTI and LTV systems at a longitudinal velocity of 100 km/h. The tracking abilities of all controllers, including the driver model, are degraded as a relatively more rapid and sophisticated control of the steering wheel is required than that at a longitudinal velocity of 60 km/h. As we predicted previously, Fig 7 also shows that the longitudinal velocity varies during the driving situation even though this value is assumed to be constant. The eMPC controller designed for the LTI system is inevitable to be vulnerable to this parameter variation. This degradation of the performance of the controller demonstrates the importance of designing the eMPC controller for LTV systems. The result of the lateral displacement error shows the improvement in the eMPC controller for the LTV system. After five seconds of the simulation, where the longitudinal velocity varies apparently enough, the error in the eMPC controller for the LTV system decreases compared to that for the LTI system. A considerable lateral error appears in the simulation of the driver model which nearly causes a collision with a traffic cone, as shown in Fig 8. The driver model and eMPC controller for the LTV system are colored by gray and red, respectively.

**Fig 9:**
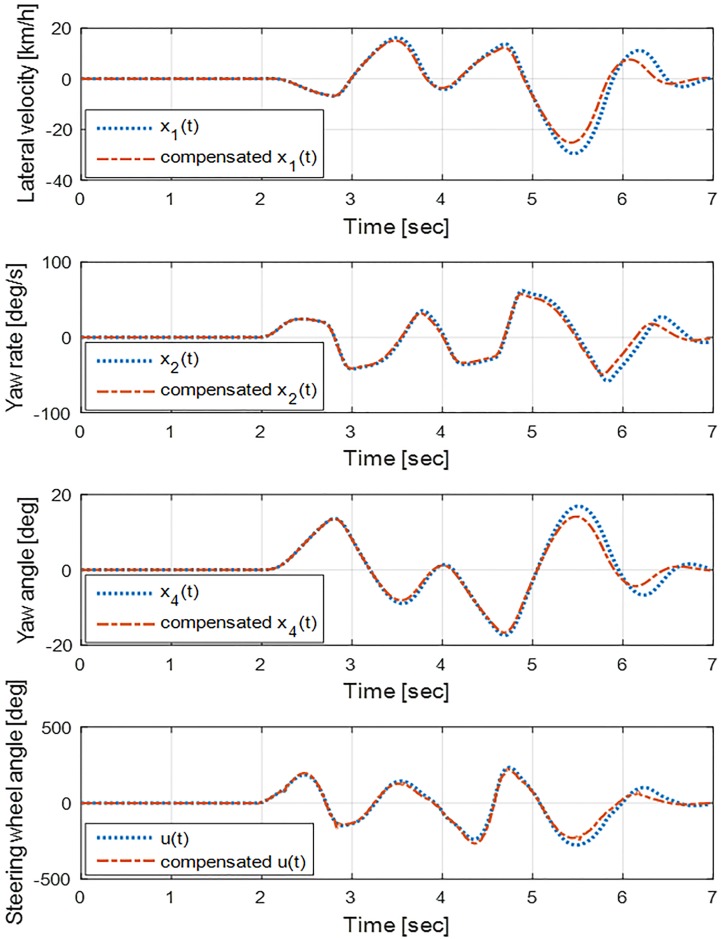
States of the vehicle model during DLC maneuver. In the implementation of the eMPC controller for the LTV system, the states are compensated regarding the parameter variation to improve the robustness of the controller. The proposed controller not only improves the tracking ability of the controller, but also enhances ride comfort as the lateral velocity is more restricted compared to the eMPC controller for the LTI system.

Fig 9 shows the state vector of the eMPC controllers for both systems, where the controller for the LTV system employs the compensated state vector. At each sampling time, the gain matrix for compensation *L*[*k*] is updated by (19) based on the recent measurment of states and inputs to consider parameter variation. As seen in Fig 9, the variation in the states of the LTV system is relatively restricted than that of the LTI system, resulting in an increase of the tracking ability. In addition, because a rapid lateral acceleration corresponds to a large sprung mass acceleration, which deteriorates the ride comfort [32], the proposed controller can also enhance the ride comfort.

Furthermore, In the actual driving situation of commercial vehicles, it frequently happens that the initial parameters change, for instance, vehicle mass due to passengers on buses or loads on trucks. Hence, robust controllers against parameter variation needs to be designed to maintain the performance of controllers for LTI systems.

## 5 Conclusion

An eMPC controller was designed for the LTV system of the vehicle. eMPC has been proposed to reduce the huge computational complexity of MPC by using an mp-QP technique. In the preliminary version of this paper, the vehicle model for the DLC maneuver is assumed to be LTI. However, such parameters, the longitudinal velocity for instance, are inevitably changed in an actual driving situation.

In this paper, we designed the eMPC controller with a simple add-on unit for the LTV system. The main contribution of this approach is that no modification is required in the critical regions of eMPC. Through the simulation results, the fulfillment of the constraints and tracking ability of the proposed controller were shown. Furthermore, it was found that the proposed controller improved ride comfort by compensating for the state vector and control input.

## 6 Future works

In the future work, we intend to conduct an actual experiment for a DLC maneuver using an electric power steering test bench to verify the applicability of the eMPC controller for autonomous driving. In addition, a more detailed analysis about the feasibility of the eMPC scheme is planned for the future work. In this paper, the eMPC controller succeeded in obtaining the control law with respect to the constraints of the vehicle and discrete-time ARE. Therefore, we intend to simulate how the eMPC controller derives the control input when the controller fails to solve the MPC problem due to more complex or harder constraints.

Moreover, because of the increased number of ECUs in electric vehicles, the time delay appeared in transmitting control or state signals has been significantly considered to cope with. Regarding this issue, in [33], a robust sliding mode controller has been designed for the systems with delay. Therefore, we also intend to employ the algorithm presented in this paper to compensate the time delay in the state and input signals.

## Supporting information

S1 Dataset Constraints for partitioning of critical regions when the prediction horizon is 40 and input horizon is 1. (XLSX)
